# Significant Enhancement of Piezoelectric Response in AlN by Yb Addition

**DOI:** 10.3390/ma14020309

**Published:** 2021-01-09

**Authors:** Kenji Hirata, Yuto Mori, Hiroshi Yamada, Masato Uehara, Sri Ayu Anggraini, Morito Akiyama

**Affiliations:** 1 Sensing System Research Center, National Institute of Advanced Industrial Science and Technology (AIST), Shuku 807-1, Tosu, Saga 841-0052, Japan; hiro-yamada@aist.go.jp (H.Y.); m.uehara@aist.go.jp (M.U.); ayu-anggraini@aist.go.jp (S.A.A.); m.akiyama@aist.go.jp (M.A.); 2 Department of Molecular and Material Sciences, Interdisciplinary Graduate School of Engineering Sciences, Kyushu University, 6-1 Kasugakoen, Kasuga, Fukuoka 816-8580, Japan; mori.yuto.nagasaki@gmail.com

**Keywords:** first-principles calculation, piezoelectric material, nitride, thin film

## Abstract

This study employs first-principles calculations to investigate how introducing Yb into aluminum nitride (AlN) leads to a large enhancement in the material’s piezoelectric response (*d*_33_). The maximum *d*_33_ is calculated to be over 100 pC/N, which is 20 times higher than that of AlN. One reason for such a significant improvement in *d*_33_ is the elastic-softening effect, which is indicated by a decrease in the elastic constant, *C*_33_. The strain sensitivity (*du/dε*) of the internal parameter, *u*, is also an important factor for improving the piezoelectric stress constant, *e*_33_. On the basis of mixing enthalpy calculations, Yb*_x_*Al*_1−x_*N is predicted to be more stable as a wurtzite phase than as a rock salt phase at composition up to *x* ≈ 0.7. These results suggest that Yb can be doped into AlN at high concentrations. It was also observed that the dielectric constant, $\varepsilon_{33}$, generally increases with increasing Yb concentrations. However, the electromechanical coupling coefficient, $k_{33}^{2}$, only increases up to *x* = 0.778, which is likely because of the relatively lower values of $\varepsilon_{33}$ within this range.

## 1. Introduction

The piezoelectric properties of aluminum nitride (AlN)-based wurtzite solid solutions have been investigated experimentally and theoretically [1,2,3,4,5,6,7,8,9,10,11]. In particular, scandium (Sc)-doped AlN (ScAlN) exhibits high piezoelectricity and has been widely considered for use in high-frequency filters, sensors, and microelectromechanical devices [12,13]. Ferroelectricity has also been observed with ScAlN, so its scope of potential applications is expected to expand [14].

Recently, an enhanced piezoelectric response was discovered in ytterbium (Yb)-doped AlN (YbAlN) [15]. However, there are very few systematic experimental reports on the phase stability and piezoelectric properties of wurtzite phase YbAlN. Although both Yb and Sc are classified as rare earth elements, it is still unclear whether the piezoelectric properties of YbAlN are equivalent to those of ScAlN. Since the piezoelectric properties of YbAlN have never been investigated in detail, it is unknown whether the observed improvement in piezoelectric response occurs via the same mechanism as previously reported AlN-based piezoelectric materials. The piezoelectric constant, *d*_33_, can be approximated as *d*_33_ ≈ *e*_33_/*C*_33_, where *e*_33_ is the piezoelectric stress constant and *C*_33_ is the elastic constant. Computational results have indicated that elastic softening (decrease of *C*_33_) can be caused by the addition of other elements into AlN [4,7,9,11]. Furthermore, the lattice parameter ratio (*c/a*) of the wurtzite structure decreases as the quantity of additional elements increases [1,2,9,16]. Changes in the piezoelectric stress constant, *e*_33_, are generally corelated with changes in the lattice parameter [17].

Typically, AlN-based piezoelectric materials are fabricated as thin films using a sputtering technique, and the solubility of the additive elements depends heavily on the sputtering conditions. The stability of the wurtzite phase is an important factor for designing AlN-based piezoelectric materials. The phase stability of rock salt (which is thermodynamically competitive with the wurtzite phase) has been evaluated by first-principles calculations [6,7,11,16], and the mixing enthalpies of the wurtzite and rock salt phases have been calculated, allowing for estimations of the stable composition of each phase [6,11]. The experimental results were generally consistent with the calculated results [1,6,11], so evaluations based on mixing enthalpy are considered reasonable. However, the thermodynamic stability of the wurtzite phase should also be evaluated for YbAlN.

In the present work, YbAlN is evaluated in terms of the lattice constant of its crystal structure, its phase stability, and the piezoelectric properties of its wurtzite phase using first-principles calculations.

## 2. Computational Methods

**Figure 1:**
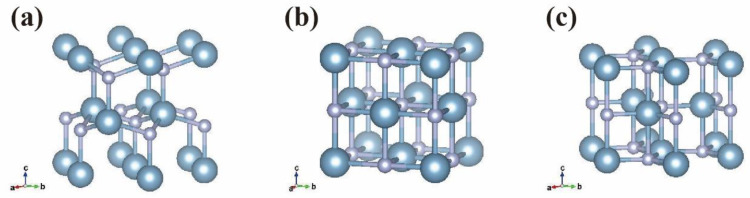
The crystal structures of (**a**) wurtzite, (**b**) rock salt, and (**c**) non-polar hexagonal phases. The blue and gray spheres represent the metal and nitrogen atoms, respectively.

The atomic configuration of YbAlN was constructed using a Special Quasirandom Structures method [18], which was implemented in Alloy Theoretic Automated Toolkit [19]. The sizes of the supercells were 3 × 3 × 2 (72 atoms), 2 × 2 × 2 (64 atoms), and 3 × 3 × 2 (72 atoms) for wurtzite, rock salt, and non-polar hexagonal phases, respectively. The crystal structure of each phase is shown in Figure 1. Yb atoms substituted Al sites in each supercell model. For structural optimization, we used the Vienna Ab initio Simulation Package (VASP) based on density functional theory [20,21]. The exchange and correlation functions were given by the generalized gradient approximation, as proposed by Perdew et al. [22]. The Blöchl’s projector-augmented wave (PAW) method was implemented as described by Kresse and Joubert [23,24]. The Monkhorst–Pack method [25] was employed for *k*-point sampling, and the meshes of the wurtzite, rock salt, and non-polar hexagonal phases were 3 × 3 × 2, 2 × 2 × 3, and 3 × 3 × 2, respectively. A cutoff energy of 500 eV was used for the plane-wave expansion. Furthermore, an on-site coulomb interaction was introduced for the ytterbium f-state. The strong correlation between localized d electrons was described using the parameter, *U*, which is defined as *U* = *U^sic^ − J*, where parameter *U^sic^* is the on-site coulomb self-interaction correction potential and *J* is the exchange integral. In this work, we used *U^sic^* = 10.329 eV and *J* = 1.349 eV [26].

To calculate the mixing enthalpy, we performed a total energy calculation after structural optimization using the tetrahedron method with Blöchl corrections. The mixing enthalpy of nitride is described in Equation (1),

(1) $$\Delta H_{mix}=H\left({\mathrm{Yb}}_{x}{\mathrm{Al}}_{1-x}N\right)-xH_{YbN}^{\mathrm{rock}\;\mathrm{salt}}-\left(1-x\right)H_{AlN}^{wurtzite}$$

where $H\left({\mathrm{Yb}}_{x}{\mathrm{Al}}_{1-x}N\right)$ is the total energy of each phase, and $H_{YbN}^{\mathrm{rock}\;\mathrm{salt}}$ and $H_{AlN}^{wurtzite}$ are the total energy of YbN (with rock salt structure) and AlN (with wurtzite structure), respectively.

Phonon and dielectric response calculations based on finite differences and density functional perturbation theory [27,28,29] were performed to calculate the elastic tensors and piezoelectric stress tensors. The piezoelectric constant, *d*_33_, is determined using Equation (2),

(2) $$d_{33}=\frac{e_{33}\left(C_{11}+C_{12}\right)-2e_{31}C_{13}}{\left(C_{11}+C_{12}\right)C_{33}-2C_{13}^{2}}$$

where *e*_ij_ and *C*_ij_ are the piezoelectric stress constant and elastic constant, respectively.

## 3. Results and Discussion

### 3.1. Evaluation of Mixing Enthalpies

**Figure 2:**
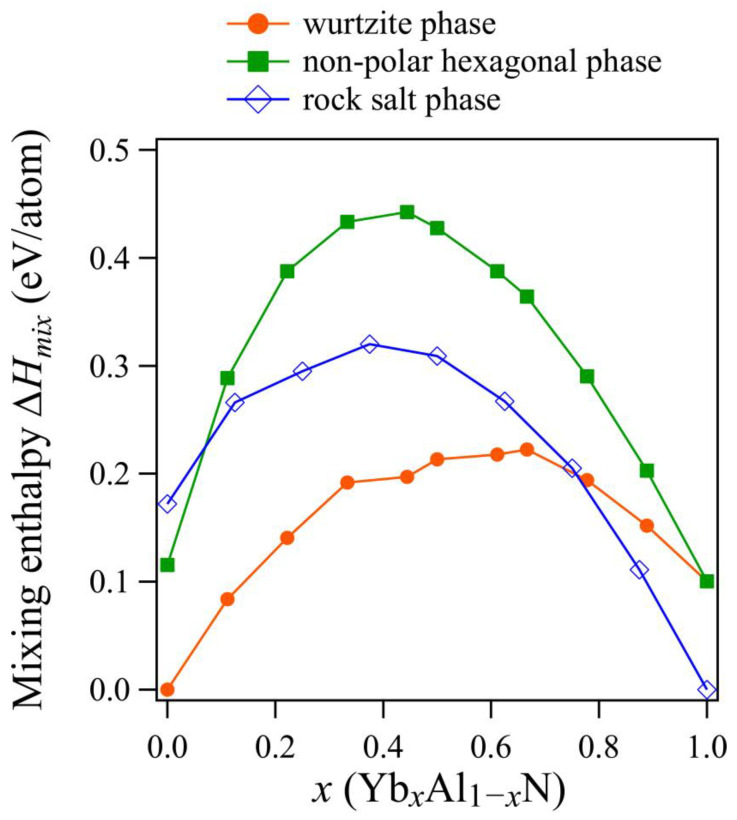
Calculated mixing enthalpies of each phase in Yb*_x_*Al_1*−x*_N. Blue, orange, and green traces correspond to the mixing enthalpies of rock salt, wurtzite, and non-polar hexagonal phases, respectively.

The formation of the YbN rock salt phase has been observed in the Yb-N binary system [30], and the thermodynamic stabilities of the wurtzite and rock salt phases are believed to compete in an AlN-YbN pseudo-binary system. The calculated mixing enthalpies of wurtzite, rock salt, and non-polar hexagonal phases are shown in Figure 2. The wurtzite phase has a smaller mixing enthalpy than the rock salt phase, and it is thermodynamically stable up to *x* = 0.7. The calculated mixing enthalpy indicates that the rock salt phase becomes more stable with the higher concentration of Yb (*x* > 0.7). To our knowledge, the phase stability of an AlN-YbN pseudo-binary system has not yet been evaluated experimentally; however, the lattice constant of rock salt YbN has been estimated as *a* = 4.79 nm [30], which is consistent with our calculated lattice constant, *a* = 4.78 nm. Generally, for thin films of AlN-based piezoelectric materials fabricated by the sputtering method, the stable phase region is estimated near the cross point of the mixing enthalpies of the rock salt and wurtzite phases [6,11]. On the basis of this concept, the wurtzite phase can potentially be obtained in a thin film until around *x* = 0.6 in Yb*_x_*Al*_1−x_*N. This tendency is similar to ScAlN [1] and MgNbAlN [2], which are reported to have a wide solubility in the wurtzite phase.

To evaluate the structural stability of the wurtzite phase, the mechanical stability was also investigated. The mechanical stability criteria for hexagonal symmetry [31] are evaluated by using the elastic stiffness constants *C*_ij_, which are described as following inequality.

(3) $$C_{44}>0,\;C_{11}>\left|C_{12}\right|,\;\left(C_{11}+2C_{12}\right)C_{33}>2C_{13}^{2}$$

The elastic stiffness constants are listed in Table 1, which were used for inequality (3). In all compositions, the elastic constants of Yb*_x_*Al_1*−x*_N satisfy the mechanical stability criteria, and the structural stability of the compounds is considered to be ensured.

The difference of mixing enthalpy between the wurtzite and the non-polar hexagonal phases tends to increase in the range of 0 ≤ *x* ≤ 0.5, and it decrease with increasing in the concentration range of 0.5 < *x* ≤ 1.0. It has been suggested that differences in mixing enthalpy affect the coordination of metal atoms in the wurtzite structure, which can lead to a drastic decrease in the lattice constant ratio, *c/a*. Therefore, it is important to discuss the relationship between the differences in mixing enthalpies and the changes in the lattice constants of the wurtzite phase in YbAlN. 

### 3.2. Evaluation of Lattice Parameters

**Figure 3:**
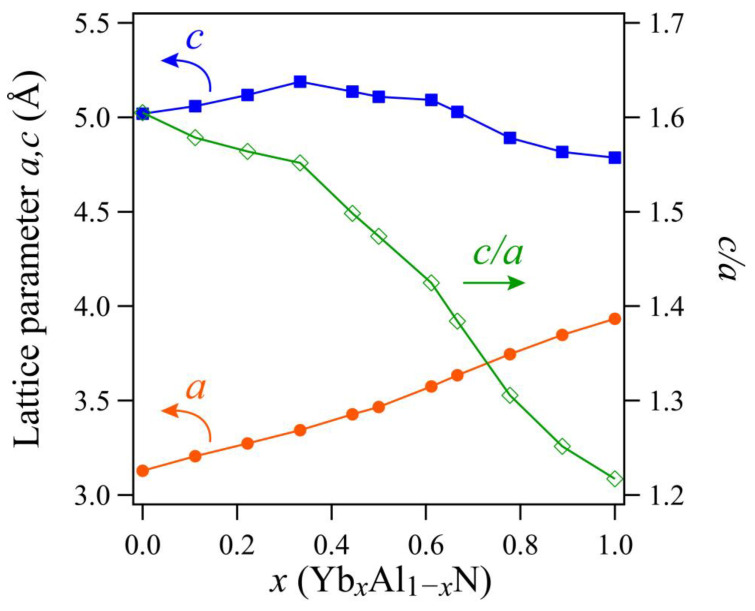
Calculated lattice parameters of Yb*_x_*Al_1*−x*_N. Orange and blue traces represent the changes to lattice parameters, *a* and *c*, respectively, and the green line denotes the ratio of lattice parameters, *c/a*.

Figure 3 shows the calculated lattice parameters of Yb*_x_*Al*_1−x_*N. The lattice constants of AlN were calculated as *a* = 3.13 nm and *c* = 5.02 nm; these values are consistent with the experimental values *a* = 3.11 nm and *c* = 4.98 nm reported by Wang et al. [32]. The lattice constant, *a*, increased monotonically as the Yb concentration increased. In contrast, the lattice constant, *c*, elongated slightly until the Yb concentration reached *x* = 0.333 and then shortened gradually as the Yb concentration increased further. Additionally, *c/a* decreased to ≈1.2 around a Yb concentration of *x* = 1.0, and the atomic configuration shifted toward a non-polar hexagonal structure. Similar behavior has been observed in other AlN-based piezoelectric materials [11,16]. Moreover, the mixing enthalpy of the non-polar hexagonal phase is close to that of the wurtzite phase at around *x* = 1.0 (see Figure 2). In the composition where *x* = 1.0, the *c/a* of the wurtzite phase is estimated around to be 1.2, which is almost equal to that of the non-polar hexagonal phase. This composition corresponds to a neighboring point between the non-polar hexagonal and wurtzite phases, which is consistent with calculations regarding ScAlN [11]. However, the composition of the neighboring point shifts to higher *x* values in YbAlN than in ScAlN (*x* = 0.8) [11].

### 3.3. Evaluation of Piezoelectric Constants

**Figure 4:**
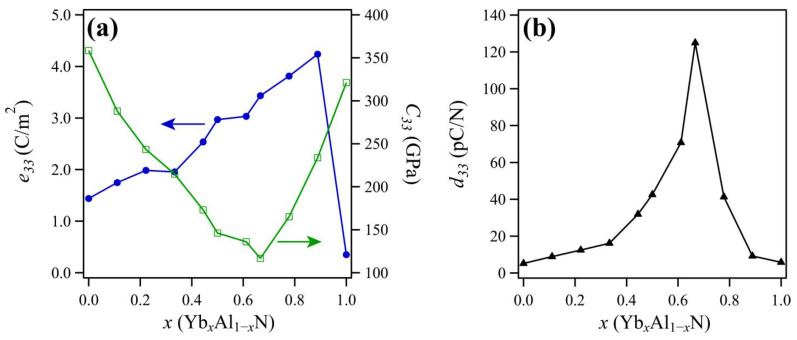
(**a**) Calculated piezoelectric stress constant, *e*_33_ (blue line), and elastic constant, *C*_33_ (green line) of Yb*_x_*Al_1*−x*_N as a function of Yb content; (**b**) calculated piezoelectric constant, *d*_33_, of Yb*_x_*Al_1*−x*_N as a function of Yb content.

Figure 4a presents the calculated piezoelectric stress constant, *e*_33_, and elastic constant, *C*_33_, and it shows that *e_33_* increases and *C*_33_ decreases with increasing concentrations of Yb. The piezoelectric constant, *d*_33_, can be approximated by *d*_33_ ≈ *e*_33_*/C*_33_, so increasing *e*_33_ and decreasing *C*_33_ lead to an increase in *d*_33_, as shown in Figure 4b. This phenomenon has also been reported for other metal-doped AlN [11]. The maximum *d*_33_ value was estimated to be over 100 pC/N, which is almost equal to the maximum theoretical *d*_33_ of ScAlN [11] and about 20 times that of AlN.

**Figure 5:**
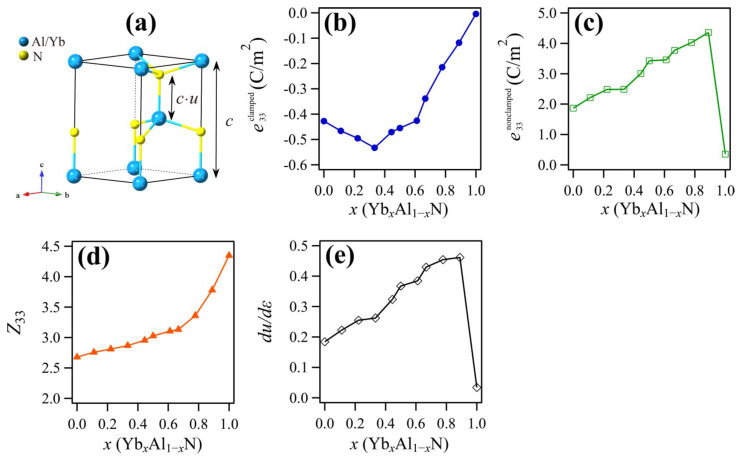
(**a**) Crystal structure of wurtzite showing the internal parameter, *u*. Blue and yellow spheres represent Al/Yb and N atoms, respectively. Other changes to the component values of *e*_33_ as a function of Yb content; (**b**) clamped *e*_33_; (**c**) nonclamped *e*_33_; (**d**) components of the nonclamped Born effective charge, *Z*_33_; and (**e**) the strain sensitivity of the internal parameter, *du/dε*.

To understand why the addition of Yb can enhance the piezoelectric stress constant, *e*_33_, we evaluated each parameter comprising *e*_33_, as shown in Equation (4),

(4) $$e_{33}=e_{33}^{clamped}+e_{33}^{nonclamped}$$

where $e_{33}^{clamped}$ is the electronic response to strain (evaluated in the equilibrium positions with fixed internal atomic coordinates). The $e_{33}^{nonclamped}$ term is the contribution from the internal atomic coordinates in response to an external strain along the *c*-direction, and this term can be defined as shown in Equation (5),

(5) $$e_{33}^{nonclamped}=\frac{4eZ_{33}}{\surd 3a^{2}}\frac{du}{d\varepsilon }$$

where *e* is the (positive) electron charge, *a* is the equilibrium lattice constant, *Z*_33_ is the Born effective charge (in units of *e*), and *ε* is the macroscopic applied strain. The internal parameter, *u*, represents the ratio between the lattice constant, *c*, and the metal–nitrogen distance along the *c*-axis in the wurtzite structure (see Figure 5a). The calculated results for these terms are shown in Figure 5b–e. The $e_{33}^{clamped}$ value decreased until the concentration of Yb reached *x* = 0.333, and it did not seem to contribute to the observed increase of *e*_33_ in this range of composition; however, at Yb concentrations higher than *x* = 0.333, $e_{33}^{clamped}$ increased and did contribute to the improvement of *e*_33_. The $e_{33}^{nonclamped}$ increased up to a Yb concentration *x* = 0.889, resulting in a value that was about twice that of AlN. Considering its magnitude, $e_{33}^{nonclamped}$ had a greater effect on the enhancement of *e*_33_ than $e_{33}^{clamped}$. Similar trends have been observed in ScAlN, so it is reasonable to believe that the displacement of ions contributes more to the enhancement of *e*_33_ than the displacement of electrons [4]. It is clear from Figure 5d that *Z*_33_ increased monotonically as the Yb concentration increased, and it reached about 1.5 times the *Z*_33_ value of AlN. Similarly, the strain sensitivity of the internal parameter (*du/dε*) increased as the Yb concentration increased. Specifically, as the Yb concentration increased from *x* = 0 to *x* = 0.889, *du/dε* increased 2.5 times, while *Z*_33_ only increased 1.5 times, indicating that the *du/dε* parameter has a stronger influence in terms of increasing $e_{33}^{nonclamped}$. A large *du/dε* value means that the displacement of atoms in the alloy is large when it experiences external strain; therefore, this factor contributes significantly to the enhancement of *e*_33_. The maximum value of *du/dε* was obtained around the composition where the *c/a* of the wurtzite phase and the non-polar hexagonal phase are the same. Similar behavior was observed in ScAlN [11], where the composition with the smallest difference between wurtzite and non-polar hexagonal phase mixing enthalpies (almost zero) also had the maximum *e*_33_. The piezoelectric properties of YbAlN are believed to be enhanced through a similar mechanism as for ScAlN. Since the lowest *C_33_* value and the highest *du/dε* value occur at the almost same Yb concentration, the increase in *du/dε* may be related to the elastic softening.

### 3.4. Evaluation of the Electromechanical Coupling Constant

**Figure 6:**
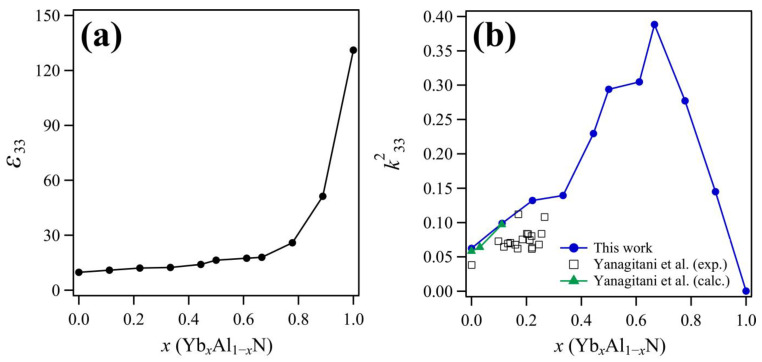
(**a**) Dielectric constant, $\varepsilon_{33}$, of Yb*_x_*Al*_1−x_*N as a function of Yb content; and (**b**) electromechanical coupling coefficient, $k_{33}^{2}$, of Yb*_x_*Al*_1−x_*N as a function of Yb content. The blue trace represents the calculated results from this work, and the black squares and green line represent the experimental and calculated results, respectively, as reported by Yanagitani et al. [15].

To verify the reliability of calculated piezoelectric properties, it is necessary to compare the calculated values with the experimental values. There is limited experimental data available regarding the piezoelectric properties of YbAlN; however, the electromechanical coupling constants ($k_{33}^{2}$) reported by Yanagitani et al. [15] can be used to confirm the reliability of our calculated results. The $k_{33}^{2}$ values were calculated using Equation (6),

(6) $$k_{33}^{2}=\frac{e_{33}^{2}}{\varepsilon_{33}C_{33}+e_{33}^{2}}$$

where $\varepsilon_{33}$ is the 33 components of the dielectric tensor, which are plotted in Figure 6a. Figure 6b shows the $k_{33}^{2}$ values calculated in this work, as well as the experimental and calculated values reported by Yanagitani et al. [15]. For pure AlN, $\varepsilon_{33}$ is calculated as 9.77, and this value is consistent with the previous computational result ($\varepsilon_{33}=9.74\;)$ [33,34]. The value of $\varepsilon_{33}$ increased monotonically as the Yb concentration in Yb*_x_*Al*_1−x_*N increased. Similarly, $k_{33}^{2}$ increased until *x* = 0.778, and then, it decreased as the Yb concentration increased further. Based on Equation (6), an increase in $\varepsilon_{33}$ leads to a decrease in $k_{33}^{2}$. The value of $\varepsilon_{33}$ was generally unaffected by the addition of Yb up to *x* = 0.778, whereas the $k_{33}^{2}$ value increased with increasing Yb concentration up to *x* = 0.778. The $k_{33}^{2}$ values for YbAlN calculated in this work are in good agreement with the computational results by Yanagitani et al. [15] in compositions with low Yb content. The experimental $k_{33}^{2}$ values determined from thin film samples tend to be lower than the calculated results obtained in this study. In general, the piezoelectric properties of a thin film are affected by (i) the crystallinity of the wurtzite phase, (ii) the orientation of the crystals, and (iii) the distribution of the polarization direction. Since the computations assume a single crystal, the calculated piezoelectric properties tend to be higher than those determined on the basis of experiments. Therefore, it is expected that the calculated results are higher than the experimental values.

On the basis of the calculated *d*_33_ and $k_{33}^{2}$ values, YbAlN is predicted to have a superior piezoelectric performance relative to AlN and to have properties comparable to ScAlN which has garnered attention as a piezoelectric material for radio frequency filters in telecommunication devices [13]. To obtain an even greater piezoelectric response with YbAlN, higher concentrations of Yb should be dissolved into wurtzite AlN. Yanagitani et al. reported that the maximum solubility of Yb in AlN is about *x* = 0.25 [15]; however, the maximum solubility of Yb is believed to vary depending on the preparation conditions of the thin film. In fact, the maximum solubility of Sc in AlN also varies depending on the preparation conditions and the substrate material [35,36]. The calculated mixing enthalpy of YbAlN (Figure 2) shows that the cross point of the wurtzite and the rock salt phases is similar to that in ScAlN (*x* = 0.6) [11,16]. Since the maximum solubility of Sc in AlN is obtained at about *x* = 0.4 (experimentally), the same level of solubility can be obtained for YbAlN by utilizing optimized preparation conditions, including the most appropriate substrate.

## 4. Conclusions

This study investigated the piezoelectric properties of Yb*_x_*Al*_1−x_*N using first-principles calculations. The wurtzite phase was revealed to be more stable than rock salt phase in compositions with up to around *x* = 0.6. Additionally, the piezoelectric stress constant, *e*_33_, increased and the elastic constant, *C*_33_, decreased with increasing Yb concentration (similar to the widely reported ScAlN), and as a result, the piezoelectric constant *d*_33_ improved. The maximum calculated *d*_33_ value was greater than 100 pC/N, which is comparable to the theoretical *d*_33_ value of ScAlN; therefore, YbAlN is also expected to be a suitable candidate for high-performance piezoelectric materials. One of the reasons for the enhancement of *d*_33_ is the contribution of elastic softening due to the decreased *C*_33_ value. The $e_{33}^{nonclamped}$ term, which is derived from the atomic displacement induced by macroscopic strain, provided the largest contribution to the enhancement of *e*_33_. The strain sensitivity (*du/dε*) of the internal parameter, *u*, exhibited the same tendency as the Yb concentration dependence of *e*_33_, which indicates that it is an important factor in *e*_33_ improvement. It was found that the dielectric constant, $\varepsilon_{33}$, generally increased with increasing Yb concentrations; however, the electromechanical coupling coefficient, $k_{33}^{2}$, increased only up to *x* = 0.778, which was likely because of the lower values of $\varepsilon_{33}$ within this range.

## Figures and Tables

**Table 1:** Elastic stiffness constants of Yb*_x_*Al_1*−x*_N.

Concentration: *x*	Elastic Stiffness Constant (GPa)				
	*C*_11_	*C*_12_	*C*_13_	*C*_33_	*C*_44_
0.000	377.6	128.4	97.9	358.5	124.3
0.111	324.1	131.4	111.7	288.0	92.2
0.222	289.0	129.3	110.6	243.3	77.0
0.333	256.5	122.0	116.7	214.8	68.5
0.444	233.7	134.3	120.6	173.1	49.3
0.500	231.5	134.2	110.5	146.1	41.7
0.611	206.7	120.6	117.9	136.0	33.5
0.667	199.5	131.9	118.4	116.7	31.5
0.778	202.3	140.8	101.3	165.2	30.1
0.889	201.3	149.5	89.0	233.7	24.1
1.000	197.9	151.7	72.5	321.1	23.2

## Data Availability

Data sharing not applicable.
